# Repeated recurrent epidermoid cyst with atypical hyperplasia: A case report and literature review

**DOI:** 10.1097/MD.0000000000008950

**Published:** 2017-12-08

**Authors:** Jialin Li, Ming Qian, Xiaoyi Huang, Li Zhao, Xinghai Yang, Jianru Xiao

**Affiliations:** a Department of Orthopedics Oncology, Changzheng Hospital; b Department of Pathology, Changhai Hospital; c Nursing School, Second Military Medical University, Shanghai, China.

**Keywords:** epidermoid cyst, malignant transformation, radiotherapy, recurrence, spine

## Abstract

**Rationale:**

Epidermoid cysts are slow-growing, benign tumor which account for less than 1% of all intraspinal tumors and epidermoid cyst with Atypical Hyperplasia is very rare. Surgical resection is the standard treatment of the tumor, but recurrence is not uncommon after incomplete resection. Inappropriate treatment can lead to repeated recurrent. Here, we reported a case of repeated recurrent epidermoid cyst with atypical hyperplasia treated with radiotherapy after surgery.

**Presenting concerns:**

A 40-year-old female presenting with intraspinal epidermoid cyst showed incomplete paraplegia in lower limbs.

**Diagnosis:**

Back pain reappeared 19 months later after surgical treatment. The patient suffered marked weakness in both limbs, along with obvious muscle atrophy and sensation deficiency of warmth and pain in left lower limb. MRI demonstrated a cystic mass with solid content and peripheral strengthen in enhanced scan.

**Interventions:**

Extended excision with intraoperative local chemotherapy and postoperative radiotherapy was performed and a dramatic reversal of symptoms was gained 4 weeks after surgery, with a total dose of 46 Gy. Postoperative pathological examination revealed epidermoid cyst with mild to moderate atypical hyperplasia.

**Outcomes:**

No acute side effects of the treatment were reported. Back pain obviously alleviated within 48 hours after surgery, while weakness and numbness of the lower limbs gradually improved and nearly disappeared in the 3-monthly follow-up visit. Until now, no recurrence is found during the 5-years follow-up.

**Lessons:**

Our study highlights that incomplete excision has led to repeated recurrent epidermoid cyst, but its complete removal with adjuvant radiotherapy has achieved remission of symptoms. Atypical hyperplasia discovered by pathological examination reminds us the possibility of malignant transformation and ensures the necessity of adequate treatment.

## 1 Introduction

Epidermoid cysts are slow-growing, benign tumor which account for < 1% of all intraspinal tumors. An intraspinal epidermoid cyst can be of either congenital or acquired origin; congenital lesions are usually associated with spinal dysraphisms such as syringomyelia, dermal sinus, and spinal bifida, while repeated lumbar puncture is the most common etiology for acquired epidermoid cysts.^[1–3]^ Intraspinal epidermoid cysts are usually located in the intradural, extrameduallary space (the outside of the dura) of the lumbosacral region with long duration of symptoms.^[4,5]^ Surgical resection is the standard treatment of the tumor, but the adherence between the capsule and spinal cord makes complete excision hard to achieve. We describe a 40-year-old female with a recurrent intraspinal epidermoid cyst and incomplete paraplegia. The lesion was unusually located in the centrums of L2–L3 and had atypical osseous destruction imaging.

## 2 Case report

### 2.1 Institutional review board statement

The study was reviewed and approved by the Ethics Committee of the Changzheng Hospital, Second Military Medical University. Informed consent was obtained from the patient.

### 2.2 Patient features

A 40-year-old female visited our center in August 2011 due to the severe weakness and numbness of lower limbs along with continuous back pain. Treatment history of the patient dated from 2008, when she accepted a surgery for an epidermoid cyst and psoas abscess in L2 with the symptoms of pain in back and left lower limb. With back pain and numbness of left lower limb, she was treated with reopening of previous surgical incision and re-excision of the cyst with posterior fixation in 14 months after the first surgery. However, back pain reappeared and catavertebral lump was found 19 months later. So she had the surgical treatment for the third time in April 2011, but the result was unsatisfied. She had no history of back injury and never underwent lumbar puncture before the first treatment (Table 1).

**Table 1:**
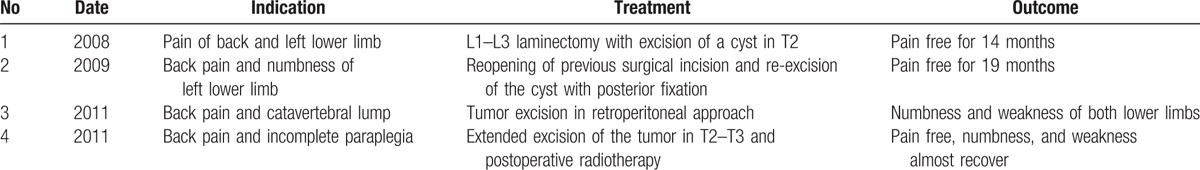
Summary of the treatment offered to the patient with indications and outcomes.

### 2.3 Physical examination

On examination, she suffered marked weakness with grade 1 power in left lower limb and grade 3 power in right lower limb, along with obvious muscle atrophy, as well as sensation deficiency of warmth and pain in left lower limb. The anus reflex disappeared, while tendon reflex was normal and babinski's sign was negative. Laboratory blood tests were uneventful.

### 2.4 Radiological findings

**Figure 1:**
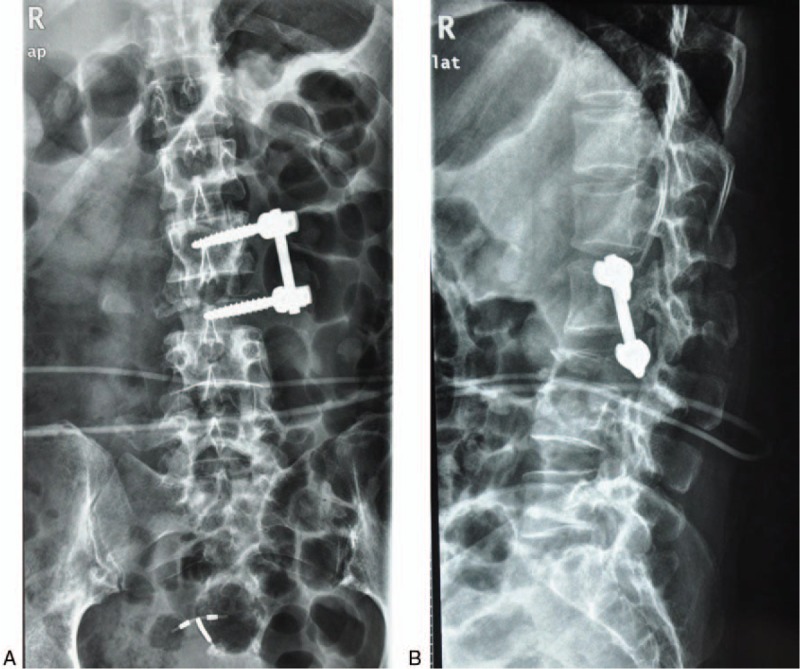
X-rays of dorsal spine revealed disappearance of lumbar physiological curvature, half-baked L2–L3 centrums, fixation in L2–L3, and swelling paravertebral soft tissue.

**Figure 2:**
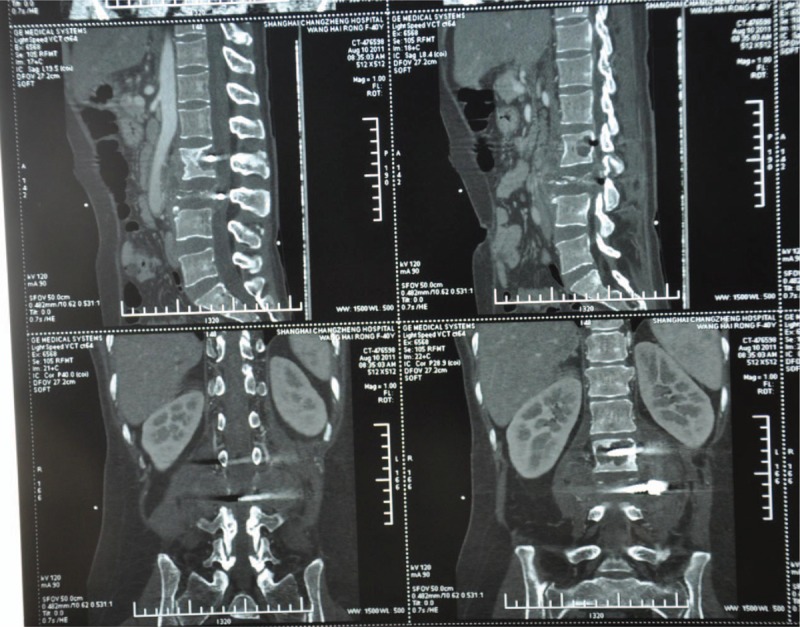
Preoperative CT images of the patient showed that osseous destruction in L2, obvious compression of centrum in L3. CT = computed tomography.

**Figure 3:**
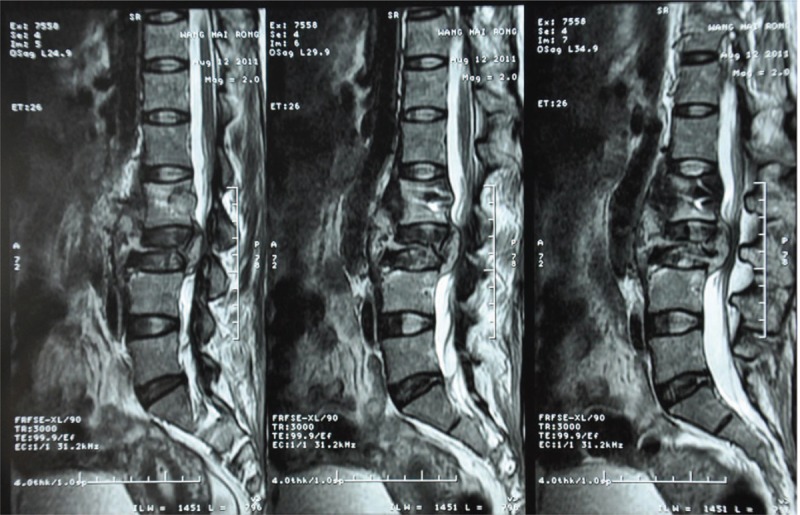
Preoperative MRI images demonstrated a cystic mass with solid content depressing the spinal cord. MRI = magnetic resonance imaging.

X-rays of dorsal spine revealed disappearance of lumbar physiological curvature, half-baked L2–L3 centrums, fixation in L2–L3, and swelling paravertebral soft tissue (Fig. 1). Computed tomography showed osseous destruction in L2, obvious compression of centrum in L3, and swelling paravertebral soft tissue (Fig. 2). Magnetic resonance imaging demonstrated a cystic mass with solid content and peripheral strengthen in an enhanced scan (Fig. 3).

### 2.5 Treatment and outcomes

**Figure 4:**
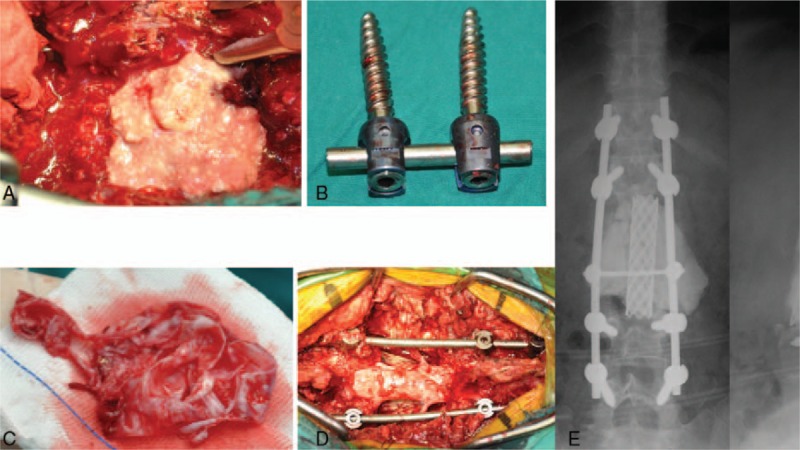
The surgical procedure of our center. (A) The tofukasu-like tissue of the cyst was observed in operation. (B) The former internal fixation screw–rod system was removed. (C) The cyst wall of the mass was removed completely. (D) There was no lesions residual. The screw–rod system reconstructed the spinal structure. (E) The x-rays after the operation showed the reconstruction was solid.

The patient underwent T12 to L5 laminectomies and a cystic mass was found in the centrums of L2–L3. Lytic damage was found in centrums and accessories of L2–L3 with heavier damage in the left side. The cystic mass was attached to the surrounding scar and soft tissue, so it was hard to be removed completely. Extended excision was performed to remove the cyst, damaged bone, and possible contaminated paravertebral soft tissue, and the content of the cyst was white, tofukasu-like tissue. The former internal fixation screw–rod system was removed. After that, the vertebral body of L2 and L3 was gross total resected by piecemeal. Deionized water 1000 mL with cisplatin 60 mg was used to soak the incision for 5 minutes to reduce possible tumor residual, then titanium mesh and screw rod system (Johnson & Johnson, New Brunswick, NJ) were used for reconstruction (Fig. 4). Intraoperative blood loss was 4500 mL and 4200 mL red cell suspension (RCS) was transfused to replenish the loss. Conventional radiotherapy irradiating L2 and L3 was performed on the patient 4 weeks after surgery, with a total dose of 46 Gy. No acute side effects of the treatment were reported. Back pain obviously alleviated within 48 hours after surgery, while weakness and numbness of the lower limbs gradually improved and nearly disappeared in the 3-month follow-up visit. The patient was kept on follow-up and no recurrence was found until March 2017 (>5 years).

### 2.6 Pathology findings

**Figure 5:**
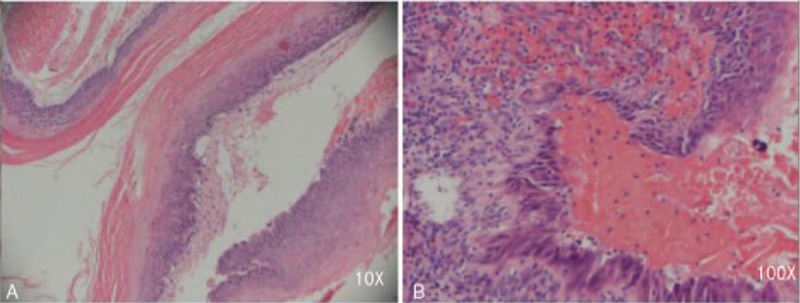
Postoperative pathology images of the patient. (A) Stratified squamous epitheliums with mild atypical hyperplasia are lined in the interior surface of the cyst capsule. Abundant stratiform keratin is located in the epithelial surface with fibrous tissue under the lining epithelium of the cyst capsule. (B) Stratified squamous epitheliums with moderate atypical hyperplasia are lined in the cyst capsule. Keratin can be found in some parts of epithelial surface. A large number of lymphocyte infiltration, necrosis, and granulation tissue necrosis reaction appear under the lining epithelium of cyst capsule.

The final histological diagnosis was an epidermoid cyst with mild to moderate atypical hyperplasia, while the tofukasu-like tissue was confirmed to be keratinized tissue (Fig. 5).

## 3 Discussion

An epidermoid cyst was first described by Cruveilhier and named “tumors perlees” (pearly tumors) in 1835.^[5]^ Epidermoid cysts within the neuraxis are rare and most of them are usually located in the intracranial region, accounting for < 1% of all intraspinal tumors.^[1,3,6]^ Pathogenesis of the tumor can be traced back to 2 possible origins: congenital or acquired.^[7]^ Congenital epidermoid cysts which account the vast majority of the tumor attribute to anomalous implantation of ectodermal cells during closure of the neural tube between the third and fifth week of embryonic life.^[3,8]^ While acquired epidermoid cysts are thought to be the result of displacement of epithelial tissue secondary to previous lumbar puncture or trauma.^[9–14]^ As for the patient, she did not have any trauma or lumbar puncture before her first surgery, so the tumor may be congenital one. However, other spinal abnormalities were also not found.

In this case, some new clinical characteristics should be noticed compared to other reported epidermoid cysts in spine.^[15,16]^ First, this case had 3 times repeated recurrences history and the patient received 4 treatments during 2008 and 2011. Recurrence of the epidermoid cyst is very rare and repeated recurrences in spine have not been reported yet.^[17]^ Only surgery was performed in the first 3 treatments, but the tumor recurrence occurred a few months later after operations. In 2011, surgery combined with radiotherapy was used to treat the third recurrence of the epidermoid cyst, in order to avoid recurrence. Second, epidermoid cyst is a benign tumor with indolent characteristic in general, while the pathological diagnosis of this case was an epidermoid cyst with atypical hyperplasia, suggesting that epidermoid cyst might have some malignant biological characteristics which required more aggressive treatment besides surgery. According to the follow-up study, the symptom of this patient almost disappeared and no recurrence occurred >5 years after radiotherapy.

Epidermoid cysts always remain asymptomatic for a relatively long period until the lesions infringe the nerves or spinal cord, along with cyst rupture which is another vital trigger. Pain and neurologic deficits such as numbness, weakness, and paralysis with long duration are the most common symptoms which are nonspecific.^[3,5,18]^ The typical imaging findings of intraspinal epidermoid cyst are hypointense on T1-weighted images and hyperintense on T2-weighted images which are consistent with the signal of the cerebrospinal fluid.^[19–21]^ Almost all the intraspinal epidermoid cysts in the literature were intradural and enlarged in the type of swelling growth, but the recurrent case was located in the centrum and increased in invasive growth which was a signal for possible malignant transformation. Imaging studies of the patient showed that lytic imaging of bone and the content of the cyst with solid change seemed to be abnormal.

Histologically, epidermoid cysts consist of lined stratified squamous epithelium supported by an outer layer of collagenous tissue. Desquamation of keratin from the epithelial lining produces soft white material rich in cholesterol crystals. The content of the cyst contains considerable fat and little cholesterol which may produce a vigorous inflammatory reaction when the cysts rupture.^[3,15,19,21–23]^ In the treatment history of the patient, psoas abscess and catavertebral lump once occurred and were thought to be the result of cyst rupture and response of surrounding tissue to the cyst content. The difference was that psoas abscess might be the result of spontaneous rupture, while a catavertebral lump was more likely caused by intraoperative spillage of the cyst content.

In spite of benign and indolent characteristic of a epidermoid cyst, complete excision is still the goal we pursue for the symptomatic lesions while asymptomatic lesions can be kept on conservative observation.^[15,23–25]^ But the goal is really hard to achieve because of the intimate adherence between the capsule and spinal cord, so subtotal excision is more often to be adopted and leaves the possibility of recurrence which may occur and quickly become symptomatic shortly after surgery.^[7,15,21,22,24,26]^ Metastatic lesions of the epidermoid cyst were never reported, while malignant transformation was very rare but there were still few intracranial cases with malignant transformation reported.^[27–31]^ The recurrent lesion of our case was the result of 3-time subtotal excisions and had some notable changes such as soluble osseous destruction, invasive growth, and solid changes of the cyst content. These changes suggest the possibility of malignant transformation, although it was not supported by the final pathological examination. But mild to moderate atypical hyperplasia let us confirm the necessity of adequate treatment and also reminded us the close follow-up of the patient.

Symptomatic recurrent lesions can be surgically treated once more but tumor excision becomes harder by formation of scar tissue resulting from the previous interventions. Even so, surgery is the most effective and curable method to remove oppression and relieve symptom for the spinal lesions. Radiotherapy once used for a repeatedly recurrent epidermoid cyst in the spine and received a relatively good result of controlling tumor growth and alleviating pain, while chemotherapy was considered useless and had never been used.^[32]^ Given that the patient had received 3 surgeries and relapsed for 3 times, we decided to adopt extended excision of the lesion with adjuvant therapies of intraoperative local chemotherapy and postoperative radiotherapy. The results were inspiring that complications of chemotherapy and radiotherapy did not appear, pain and neurologic deficit recovered well, and no recurrence has been found until now.

## 4 Conclusion

An epidermoid cyst is a rare benign tumor which can occur and cause severe neurological deficits. Complete excision is the standard treatment but is hard to reach, so recurrences after subtotal excision are not uncommon. Special attention should be paid to the treatment for recurrent lesions because repeated recurrence may lead to malignant transformation. Complete excision at the utmost with the help of postoperative radiotherapy can serve as an ideal choice.

## References

[1] Amato VG Assietti R Arienta C Intramedullary epidermoid cyst: preoperative diagnosis and surgical management after MRI introduction. Case report and updating of the literature. J Neurosurg Sci 2002;46:122–6. 12690335

[2] Lunardi P Missori P Gagliardi FM Long-term results of the surgical treatment of spinal dermoid and epidermoid tumors. Neurosurgery 1989;25:860–4. 2601815 10.1097/00006123-198912000-00002

[3] Roux A Mercier C Larbrisseau A Intramedullary epidermoid cysts of the spinal cord. Case report. J Neurosurg 1992;76:528–33. 1738035 10.3171/jns.1992.76.3.0528

[4] Bloomer CW Ackerman A Bhatia RG Imaging for spine tumors and new applications. Top Magn Reson Imaging 2006;17:69–87. 17198224 10.1097/RMR.0b013e31802bb38f

[5] Zavanone M Guerra P Rampini PM A cervico-dorsal intramedullary epidermoid cyst. Case report and review of the literature. J Neurosurg Sci 1991;35:111–5. 1757803

[6] Babayev R Abbasov B Eksi MS Thoracic intramedullary epidermoid cyst-timely fashion diagnosis and treatment. Child's Nerv Syst 2015;31:793–6. 25681950 10.1007/s00381-015-2625-6

[7] Scarrow AM Levy EI Gerszten PC Epidermoid cyst of the thoracic spine: case history. Clin Neurol Neurosurg 2001;103:220–2. 11714565 10.1016/s0303-8467(01)00156-1

[8] Chandra PS Manjari T Devi BI Intramedullary spinal epidermoid cyst. Neurol India 2000;48:75–7. 10751819

[9] Baba H Wada M Tanaka Y Intraspinal epidermoid after lumbar puncture. Int Orthop 1994;18:116–8. 8039955 10.1007/BF02484422

[10] Gardner DJ O’Gorman AM Blundell JE Intraspinal epidermoid tumour: late complication of lumbar puncture. CMAJ 1989;141:223–5. 2752348 PMC1269411

[11] Pear BL Iatrogenic intraspinal epidermoid sequestration cysts. Radiology 1969;92:251–4. 5765928 10.1148/92.2.251

[12] Park MH Cho TG Moon JG Iatrogenic intraspinal epidermoid cyst. Korean J Spine 2014;11:195–7. 25346768 10.14245/kjs.2014.11.3.195 PMC4206976

[13] Singh K Pandey S Gupta PK Acquired dorsal intraspinal epidermoid cyst in an adult female. Surg Neurol Int 2016;7 (suppl 3):S67–9. 26904369 10.4103/2152-7806.174890 PMC4743265

[14] Sheng HS Lin J Wang HO Spinal epidermoid cyst formation after spinal fracture operation: a case report. Turk Neurosurg 2013;23:800–2. 24310466 10.5137/1019-5149.JTN.5216-11.1

[15] Fereydoonian NA Bakhti S Fereshtehnejad SM Intramedullary thoracic spine epidermoid cyst with myelopathic presentations: a report of a rare case. Clin Neurol Neurosurg 2013;115:841–3. 22959213 10.1016/j.clineuro.2012.08.002

[16] Yin H Zhang D Wu Z Surgery and outcomes of six patients with intradural epidermoid cysts in the lumbar spine. World J Surg Oncol 2014;12:50. 24589060 10.1186/1477-7819-12-50 PMC3975861

[17] Shah A Patil M Goel A Recurrent craniospinal epidermoid: a case report. J Craniovertebr Junction Spine 2016;7:59–61. 27041888 10.4103/0974-8237.176627 PMC4790151

[18] Biliciler B Vatansever M Fuat Erten S A huge intramedullary epidermoid cyst: mimicking cauda equina ependymoma. Diagnostic failure of myelography and myelo-CT. J Neurosurg Sci 1996;40:149–52. 9049900

[19] Munshi A Talapatra K Ramadwar M Spinal epidermoid cyst with sudden onset of paraplegia. J Cancer Res Ther 2009;5:290–2. 20160364 10.4103/0973-1482.59913

[20] Teo BT Lin CC Chiou TL Unusual magnetic resonance characteristics of a cerebellopontine angle epidermoid cyst with upper cervical spinal canal extension. J Clin Neurosci 2006;13:781–4. 16723231 10.1016/j.jocn.2005.08.011

[21] Yen CP Kung SS Kwan AL Epidermoid cysts associated with thoracic meningocele. Acta Neurochir 2008;150:305–8. discussion 308-309. 18193152 10.1007/s00701-007-1398-4

[22] Gonzalvo A Hall N McMahon JH Intramedullary spinal epidermoid cyst of the upper thoracic region. J Clin Neurosci 2009;16:142–4. 19013801 10.1016/j.jocn.2008.04.017

[23] Kumar A Singh P Jain P Intramedullary spinal epidermoid cyst of the cervicodorsal region: A rare entity. J Pediatr Neurosci 2010;5:49–51. 21042510 10.4103/1817-1745.66675 PMC2964782

[24] Fleming C Kaliaperumal C O'Sullivan M Recurrent intramedullary epidermoid cyst of conus medullaris. BMJ Case Rep 2011;2011:pii: bcr1120115090. 10.1136/bcr.11.2011.5090 PMC3238133 22669964

[25] Ogden AT Khandji AG McCormick PC Intramedullary inclusion cysts of the cervicothoracic junction. Report of two cases in adults and review of the literature. J Neurosurg Spine 2007;7:236–42. 17688066 10.3171/SPI-07/08/236

[26] Cataltepe O Berker M Akalan N A giant intramedullary spinal epidermoid cyst of the cervicothoracic region. Pediatr Neurosurg 2004;40:120–3. 15367801 10.1159/000079853

[27] Chon KH Lee JM Koh EJ Malignant transformation of an epidermoid cyst in the cerebellopontine angle. J Korean Neurosurg Soc 2012;52:148–51. 23091675 10.3340/jkns.2012.52.2.148 PMC3467374

[28] Hamlat A Hua ZF Saikali S Malignant transformation of intra-cranial epithelial cysts: systematic article review. J Neurooncol 2005;74:187–94. 16193391 10.1007/s11060-004-5175-4

[29] Link MJ Cohen PL Breneman JC Malignant squamous degeneration of a cerebellopontine angle epidermoid tumor. Case report. J Neurosurg 2002;97:1237–43. 12450053 10.3171/jns.2002.97.5.1237

[30] Sawan B Vital A Loiseau H Squamous cell carcinoma developing in an intracranial prepontine epidermoid cyst. Ann Pathol 2000;20:258–60. 10891726

[31] Tamura K Aoyagi M Wakimoto H Malignant transformation eight years after removal of a benign epidermoid cyst: a case report. J Neurooncol 2006;79:67–72. 16583265 10.1007/s11060-005-9117-6

[32] Bretz A Van den Berge D Storme G Intraspinal epidermoid cyst successfully treated with radiotherapy: case report. Neurosurgery 2003;53:1429–31. discussion 1431-1422. 14633311 10.1227/01.neu.0000093828.70768.40

